# The Collagen-Based Medical Device MD-Tissue Acts as a Mechanical Scaffold Influencing Morpho-Functional Properties of Cultured Human Tenocytes

**DOI:** 10.3390/cells9122641

**Published:** 2020-12-08

**Authors:** Filippo Randelli, Patrizia Sartori, Cristiano Carlomagno, Marzia Bedoni, Alessandra Menon, Elena Vezzoli, Michele Sommariva, Nicoletta Gagliano

**Affiliations:** 1 Hip Department (CAD) Gaetano Pini—CTO Orthopedic Institute, Università degli Studi di Milano, Piazza Cardinal Ferrari 1, 20122 Milan, Italy; filippo.randelli@fastwebnet.it; 2 Department of Biomedical Sciences for Health, Università degli Studi di Milano, via Mangiagalli 31, 20133 Milan, Italy; patrizia.sartori@unimi.it (P.S.); ale.menon@me.com (A.M.); elena.vezzoli@unimi.it (E.V.); michele.sommariva@unimi.it (M.S.); 3 IRCCS Fondazione Don Carlo Gnocchi ONLUS, via Capecelatro 66, 20148 Milan, Italy; ccarlomagno@DONGNOCCHI.IT (C.C.); mbedoni@DONGNOCCHI.IT (M.B.); 4 U.O.C. 1° Clinica Ortopedica, ASST Centro Specialistico Ortopedico Traumatologico Gaetano Pini-CTO, Piazza Cardinal Ferrari 1, 20122 Milan, Italy

**Keywords:** tendon, tenocytes, tendinopathy, collagen turnover, mechanotransduction, actin cytoskeleton, YAP/TAZ, medical device

## Abstract

Mechanotransduction is the ability of cells to translate mechanical stimuli into biochemical signals that can ultimately influence gene expression, cell morphology and cell fate. Tenocytes are responsible for tendon mechanical adaptation converting mechanical stimuli imposed during mechanical loading, thus affecting extracellular matrix homeostasis. Since we previously demonstrated that MD-Tissue, an injectable collagen-based medical compound containing swine-derived collagen as the main component, is able to affect tenocyte properties, the aim of this study was to analyze whether the effects triggered by MD-Tissue were based on mechanotransduction-related mechanisms. For this purpose, MD-Tissue was used to coat Petri dishes and cytochalasin B was used to deprive tenocytes of mechanical stimulation mediated by the actin cytoskeleton. Cell morphology, migration, collagen turnover pathways and the expression of key mechanosensors were analyzed by morphological and molecular methods. Our findings confirm that MD-Tissue affects collagen turnover pathways and favors cell migration and show that the MD-Tissue-induced effect represents a mechanical input involving the mechanotransduction machinery. Overall, MD-Tissue, acting as a mechanical scaffold, could represent an effective medical device for a novel therapeutic, regenerative and rehabilitative approach to favor tendon healing in tendinopathies.

## 1. Introduction

Tendinopathy is a chronic and painful condition affecting tendons, characterized by histological modifications such as hypercellularity, neovascularization, loss of collagen fibril organization, increased proteoglycan and glycosaminoglycan contents and increased non-collagen extracellular matrix components [1,2]. The therapeutic approach for tendinopathy includes rest, ice-packs, non-steroidal anti-inflammatory drugs (NSAIDs), physiotherapy, local corticosteroid injections or biological and regenerative therapies using platelet-rich plasma (PRP) or hyaluronic acid [3]. However, treatment of tendinopathy remains a clinical unmet need, since the available treatments did not show to have a strong efficacy and no long-term benefits were reported [2,4,5]. Therapeutic strategies are also needed in veterinary medicine to especially treat equine tendon lesions and musculoskeletal disorders [6,7,8]. MD-Tissue (MD) is an injectable collagen-based medical compound containing swine-derived collagen as the main component. Swine collagen has high biocompatibility with human collagen, with a very low risk of adverse effects when used in different medical applications, and it was also used to prepare collagen-based skin-like scaffolds [9]. Indeed, clinical studies reported that MD-Knee, a collagen-based medical compound very similar in terms of composition to MD, is well tolerated, and no systemic adverse events or septic complications were observed when utilized on patients [10,11]. Therefore, MD may have the potential to be used to treat tendinopathy. Moreover, since it can be utilized alone or in association with other therapeutic agents, and the lower cost compared to hyaluronic acid could favor its wider use, it may offer some advantages compared to other biological agents.

Tenocytes are specialized fibroblasts in tendon connective tissue, responsible for tendon extracellular matrix (ECM) remodeling by influencing the turnover pathways of type I collagen (COL-I), the main component of tendon ECM [12,13,14]. Tendons are interposed between muscles and bones and transfer forces generated by muscle contraction to the skeleton. Mechanical forces acting on tendons influence their metabolic activity and the expression of genes and proteins involved in ECM remodeling of tenocytes that play key roles acting as mechanosensors [13,15,16].

Mechanotransduction is the ability of cells to translate mechanical stimuli into biochemical signals that can ultimately influence gene expression, cell morphology and cell fate. Mechanotransduction allows cells to respond to external forces and to interpret the mechanical characteristics of the ECM. In this way, tenocytes can timely adapt to the continuous dynamic modifications of the ECM by remodeling it [17,18]. Recently, we analyzed the in vitro effect of MD on human tenocytes [19]. We focused our attention on collagen turnover pathways, in order to describe the molecular mechanisms triggered by this medical compound and to understand how it can affect tenocytes’ biological properties to favor tendon homeostasis and repair [19]. In fact, in that study, we reported that MD was able to stimulate COL-I biosynthesis, secretion and maturation and to induce tenocyte proliferation and migration. Since tenocytes act as mechanosensors and it was demonstrated that MD is able to affect collagen turnover pathways and cell migration, the aim of this study was to analyze whether the effects triggered by MD were based on mechanotransduction-related mechanisms.

## 2. Materials and Methods

### 2.1. Samples

**Figure 1:**
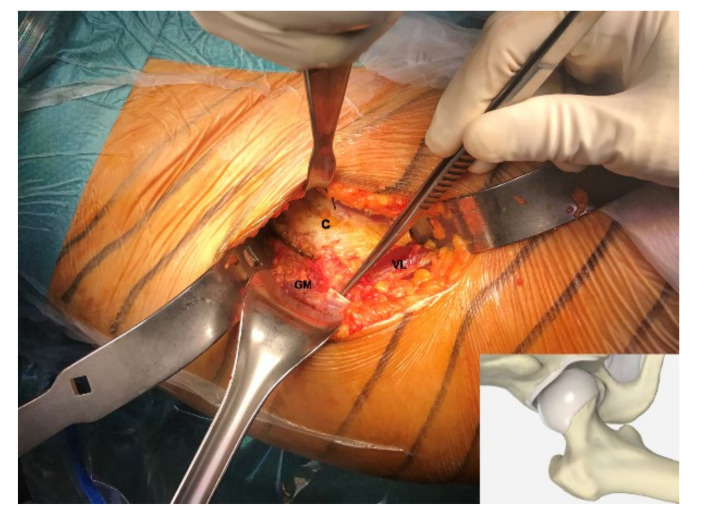
Harvesting a small fragment from the Gluteus Minimus (GM) tendon, indicated by the surgical forceps, during a total hip replacement through an anterior approach. The small fragment is collected at the mid-tendon substance, the white region with the typical structure of the dense regular connective tissue. The hip capsule (C) has been isolated and the Vastus Lateralis (VS) is visible at the bottom of the surgical field.

Fragments from the human Gluteus Minimus tendon were obtained from 4 patients (mean age 62.25 ± 4.57 years, 2 males and 2 females) undergoing total hip replacement through an anterior approach but without any gluteal tendon pathology (Figure 1). Patients diagnosed with great trochanter tendinopathy, affected by genetic collagen disorders, or patients diagnosed with spondyloarthritis with involvement of the affected hip or affected by psoriatic arthritis were excluded from the study, as well as drug- and alcohol-addicted patients, pregnant or breastfeeding women and patients affected by diabetes mellitus or who had taken fluoroquinolones within 30 days before the surgery.

For each collected tendon, the mid-substance, the region with the typical structure of the dense regular connective tissue, was isolated and analyzed.

All subjects gave their informed consent for inclusion in the study. The study was conducted in accordance with the Declaration of Helsinki, and the protocol was approved by the local ethics committee (San Raffaele Hospital Ethical Committee, Milan, Italy) of the coordinating institution (IRCCS Policlinico San Donato, Milan, Italy) (63/INT/2017).

### 2.2. Cell Cultures

**Figure 2:**
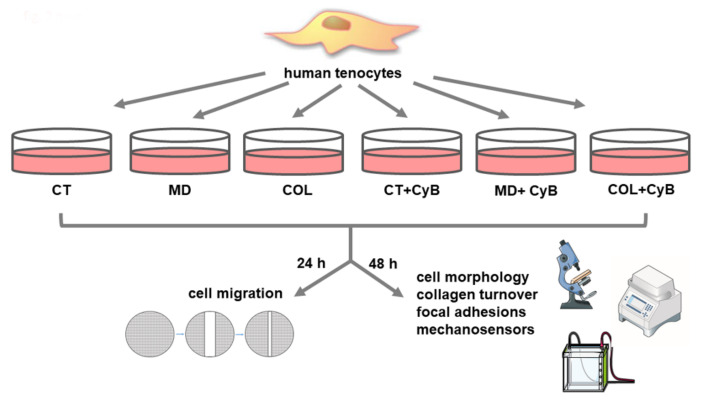
Diagram summarizing the experimental groups and the experimental design used in this study.

Tendon fragments were collected and immediately washed in sterile PBS. They were plated in T25 flasks and incubated in Dulbecco’s Modified Eagle Medium (DMEM) (Euroclone, Pero, Milan, Italy) supplemented with 10% heat-inactivated fetal bovine serum (FBS) (Gibco, Life Technologies, Monza, Italy) and antibiotics (100 U/mL penicillin, 0.1 mg/mL streptomycin) (Euroclone), at 37 °C in a humidified atmosphere containing 5% CO_2_. When tenocytes grew out from the explant, they were harvested and subcultured in T75 flasks. Human tenocytes derived for each subject were cultured in duplicate. For morphological, functional and molecular evaluations, confluent tenocytes were cultured in 6-well multi-well plates at the fifth passage, adding ascorbic acid (200 µM) to DMEM to preserve collagen synthesis, and harvested after 48 h. A diagram summarizing the experimental design of the study is shown in Figure 2.

### 2.3. Coating with MD-Tissue or Collagen

MD (100 µg/2 mL ampoules) and collagen (COL) were kindly provided by Guna (Milan, Italy). COL is the collagen of swine origin, the principal constituent of MD, that also contains ascorbic acid, magnesium gluconate, pyridoxin hydrochloride, riboflavin, thiamine hydrochloride, NaCl and water as excipients. MD or COL (50 µg/mL) were used to obtain a thin coating on 6-well multi-well plates as previously described [19]. After an incubation of at least 3–4 h at room temperature to obtain collagen adhesion to the plastic, excess fluid was removed from the coated surface and the multi-well plate was dried under the laminar flux hood. Coated plastic was immediately used or stored at 4 °C. Cells cultured on MD-Tissue or COL were compared with cells grown on uncoated cell culture plastic, used as untreated controls (CT).

### 2.4. Cytochalasin Administration

To understand if MD exerts its effect on tenocytes by a mechanical stimulation, cells were treated with 10 µM cytochalasin B (CyB) (Santa Cruz Biotechnology, Heidelberg, Germany) which inhibits actin filaments polymerization. The dose of CyB used to treat tenocytes was chosen according to the literature [20]. Moreover, different doses were tested to evaluate the possible microfilament modifications leading to cytoskeleton injury.

### 2.5. Scanning Electron Microscopy

The coating containing MD and COL was observed with a scanning electron microscope (SEM) to detect the presence of collagen fibrils/fibers and their alignment. For this purpose, the samples were fixed with 2% glutaraldehyde and 2% paraformaldehyde buffered with 0.1 M sodium cacodylate (pH 7.3) for 1 h at room temperature. After fixation, they were rinsed three times with 0.2 M sodium cacodylate buffer (pH 7.3) for 10 min and post-fixed with 1% osmium tetroxide (OsO4) in the same buffer for 1 h on ice. Samples were rinsed twice with bi-distilled water and gradually dehydrated by consecutive 10-min incubations in 20%, 30%, 40%, 50%, 70%, 80%, 90% and 100% ethanol, followed by chemical drying with 50% (*v*/*v*) ethanol-hexamethyldisilazane (HMDS) and 100% HMDS that was air-dried overnight at room temperature. All the reagents were purchased from Electron Microscopy Sciences (Hatfield, PA, USA). Before SEM imaging, samples were mounted on 12-mm specimen stubs using double-sided carbon tape and gold coated with a 20 nm-thick film using a Polaron E5100 sputter coater. The SEM imaging was performed by a JEOL JSM-840A (Tokyo, Japan), operating at 15 kV and acquiring the secondary electron signal by an Everhart-Thornley (ET) in-chamber detector.

### 2.6. Raman Spectroscopy

Raman spectroscopy was used to analyze the coating containing MD or COL. Raman spectra were acquired using an Aramis Raman microscope (Horiba Jobin Yvon, France) equipped with a laser source operating at 532 nm. All the materials were analyzed in the 400–1800 cm^−1^ range, with a spectral resolution of 0.8 cm^−1^ and accumulation time of 30 s repeated on the same point for 2 accumulations. The acquisition delay time was maintained at 2 s in order to prevent the formation of artifact spectra. Before each analysis, the instrument was calibrated on the reference band of silicon at 520.7 cm^−1^. All the samples were analyzed using a line-focused map (at least 25 points) centered using 10x, 50x and 100x objectives (Olympus, Tokyo, Japan). A laser grating of 1800, with hole at 400 and slit at 200, was used. Sample preparation was conducted depositing a 5 µL drop on a Calcium Fluoride (CaFl_2_) disk, dried overnight at room temperature. The data processing procedure was performed following and adapting the protocol reported by Carlomagno et al. [21]. Briefly, all the spectra were fit with a third-degree polynomial baseline, considering 68 baseline points, and consecutively normalized by a unit vector. A second-degree Savitzky–Golay smoothing was applied in order to reduce noise and non-informative spikes present in the resultant spectra. All the procedures described were performed using the Raman integrated software LabSpec6 (Horiba Jobin Yvon, France) and Origin2018 (OriginLab, Northampton, MA, USA).

### 2.7. Immunofluorescence Analysis

For fluorescence microscopy, tenocytes were cultured on 12-mm diameter round coverslips uncoated or coated with MD or COL into 24-well culture plates, with or without CyB, as previously described [22]. For vinculin detection, cells were incubated for 1 h at room temperature with the mouse monoclonal antibody anti-vinculin (1:500 in PBS, clone VIN-11-5, Biotechne, Milan, Italy) and with the secondary antibody anti-mouse/Alexa488 (1:500, Life Technologies, Carlsbad, CA, USA). In order to analyze the actin cytoskeleton, cells were incubated with 50 µM rhodamine-phalloidin (Sigma-Aldrich, St. Louis, MO, USA).

To assess YAP/TAZ nuclear or cytoplasmic localization, cells were incubated with a rabbit anti-YAP/TAZ antibody (D24E4, 1:400, Cell Signaling, Danvers, MA, USA) and an anti-rabbit/Alexa488 (1:500, Cell Signaling, Danvers, MA, USA).

Finally, cells on coverslips were incubated with DAPI (1:100.000, Sigma Aldrich) for 15 min and mounted onto glass slides using Mowiol. Cells were analyzed and imaged by a WD THUNDER Imager Tissue 3D (Leica Microsystems CMS GmbH, Wetzlar, Germany).

### 2.8. Real-Time PCR

Cells were harvested and total RNA was isolated (Tri-Reagent, Sigma, Italy). One µg of total RNA was reverse-transcribed in 20 µL final volume of reaction mix (Biorad, Segrate, Milan, Italy). Gene expression for long lysyl hydroxylase 2 (LH2b), tissue inhibitor of matrix metalloproteinase 1 (TIMP-1), focal adhesion kinase (FAK) and paxillin (PAX) was analyzed by real-time RT-PCR in samples run in triplicate. Glyceraldehyde 3-phosphate dehydrogenase (GAPDH) was used as endogenous control to normalize the differences in the amount of total RNA in each sample. The primers sequences were the following: GAPDH: sense CCCTTCATTGACCTCAACTACATG, antisense TGGGATTTCCATTGATGACAAGC; LH2b: sense CCGGAAACATTCCAAATGCTCAG, antisense GCCAGAGGTCATTGTTATAATGGG; TIMP-1: sense GGCTTCTGGCATCCTGTTGTTG, antisense AAGGTGGTCTGGTTGACTTCTGG; FAK: sense GTCTGCCTTCGCTTCACG, antisense GAATTTGTAACTGGAAGATGCAAG; and PAX: sense CAGCAGACACGCATCTCG, antisense GAGCTGCTCCCTGTCTTCC. Each sample was analyzed in triplicate in a Bioer LineGene 9600 thermal cycler (Bioer, Hangzhou, China). The cycle threshold (Ct) was determined and gene expression levels relative to that of GAPDH were calculated using the ΔC_T_ method.

### 2.9. Slot Blot

Collagen type I (COL-I) and matrix metalloproteinase (MMP)-1 protein levels secreted by tenocytes in serum-free cell supernatants were analyzed by slot blot analysis, as previously detailed [18]. Membranes were incubated for 1 h at room temperature with primary monoclonal antibodies to COL-I (1:1000 in TBST) (Sigma-Aldrich, Milan, Italy) or MMP-1 (1 µg/mL in TBST) (Millipore, Milan, Italy). Immunoreactive bands were revealed by the Amplified Opti-4CN substrate (Amplified Opti-4CN, Bio Rad, Segrate, Milan, Italy) and quantification was obtained after densitometric scanning of immunoreactive bands (UVBand, Eppendorf, Italy).

### 2.10. Western Blot

Cells were harvested and lysed in Tris-HCl 50 mM pH 7.6, 150 mM NaCl, 1% Triton X-100, 5 mM EDTA, 1% Sodium Dodecyl Sulphate (SDS), proteases inhibitors and 1 mM sodium orthovanadate. After a 30-min incubation in ice, lysates were centrifuged at 14,000× *g* for 10 min at 4 °C. Cell lysates (15 µg of total proteins) were run on 10% SDS–polyacrylamide gel, separated under reducing and denaturing conditions at 80 V according to Laemmli and transferred at 90 V for 90 min to a nitrocellulose membrane in 0.025 M Tris, 192 mM glycine and 20% methanol, pH 8.3. For VNC analysis, membranes were incubated for 1 h at room temperature with the monoclonal antibody anti-VNC (1:2000) (clone VIN-11-5, Biotechne, Milan, Italy) and, after washing, in horseradish peroxidase (HRP)-conjugated rabbit anti-mouse antibody (1:6000 dilution, Sigma Aldrich). Immunoreactive bands were revealed using the Opti-4CN substrate (Bio Rad).

For YAP/TAZ evaluation, membranes were incubated with the following antibodies (Cell Signaling Technology, USA): YAP (D8H1X) XP^®^ Rabbit mAb, p-YAP (S109) Rabbit Ab, TAZ (D3I6D) Rabbit mAb and p-TAZ (S89) (E1X9C) Rabbit mAb. After the incubation with a horseradish peroxidase (HRP)-conjugated goat anti-rabbit antibody (1:20000 dilution, Cell Signaling), immunoreactive bands were revealed using the Amplified Opti-4CN (Bio Rad).

To confirm equal loading, membranes were reprobed by a monoclonal antibody to α-tubulin (1:2000 dilution, Sigma Aldrich).

### 2.11. SDS-Zymography

MMP-2 levels and activity were analyzed in serum-free culture supernatants (5 μg of total protein per sample) in tenocytes cultured for 48 h by SDS-zymography on 10% polyacrylamide gels co-polymerized with 1 mg/mL type I gelatin. The gels were run at 4 °C and, after SDS-PAGE, they were washed twice in 2.5% Triton X-100 for 30 min each and incubated overnight in a substrate buffer at 37 °C (Tris-HCl 50 mM, CaCl_2_ 5 mM, NaN_3_ 0.02%, pH 7.5). After staining and destaining the gels, MMP gelatinolytic activity was detected as clear bands on a blue background after staining the gels with Coomassie brilliant blue R250. Clear bands were quantified by densitometric scanning (UVBand, Eppendorf, Italy).

### 2.12. Wound Healing Assay

Cell migration of tenocytes was analyzed by a wound healing assay [23] in CT-, MD- or COL-coated 6-well multi-well plates. The “scratch” was obtained in confluent tenocytes using a p 200 pipet tip. After washing with DMEM to remove cell debris, multi-well plates were incubated in serum-free DMEM at 37 °C and observed under an inverted microscope. Migration was evaluated by measuring the closure of the wound at 0 and 24 h.

Digital images were captured by a digital camera at different time points (0 and 24 h), and the size of the “scratch” was measured to assess the migration potential, expressed as a % compared with the 0 h time point.

### 2.13. Statistical Analysis

Data were obtained from two replicate experiments for each of the subjects-derived cell lines cultured in duplicate and were expressed as mean ± standard deviation (SD). Statistical analysis was performed by *t*-test to compare untreated vs. CyB-treated samples cultured on the same substrate and ANOVA followed by Tukey’s multiple comparisons test using GraphPad Prism v 5.0 software (GraphPad Software Inc., San Diego, CA 92108, USA). Differences associated with *p* values lower than 5% were considered statistically significant.

## 3. Results

### 3.1. Analysis and Characterization of the Coating

**Figure 3:**
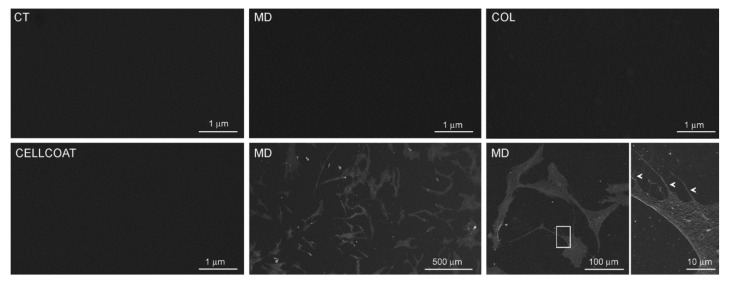
Scanning electron microscopy (SEM) images of Petri dish uncoated (CT) or coated with MD-Tissue (MD) or collagen (COL) solution. An SEM image of a commercial Petri dish coated with Type I collagen (CELLCOAT Type I Collagen—Greiner bio-one cod.628950) is also shown. SEM images of human tenocytes cultured on an MD Tissue-coated Petri dish at low (left) and high magnification (right); in the inset at higher magnification, the thin flattened processes extending from the cell body (arrowheads) are visible. The scale bar is shown in the bottom right corner of each image.

The presence and the characteristics of the coating obtained using MD or COL were analyzed by scanning electron microscopy (SEM). We did not detect collagen fibrils in Petri dishes coated with MD or COL (Figure 3), compared to CT. As a control, we compared MD- and COL-coated Petri dishes with a commercially available Petri dish coated with Type I collagen (CELLCOAT Type I Collagen—Greiner bio-one cod.628950), in which the presence of the coating resulted undetectable at SEM as well (Figure 3).

To understand if the coating influences cell alignment, cells were grown on 12-mm diameter coverslips coated with MD: SEM analysis confirmed that collagen fibrils are undetectable and that the coating does not induce cell alignment. Indeed, cells were arranged without any preferential direction (Figure 3).

**Figure 4:**
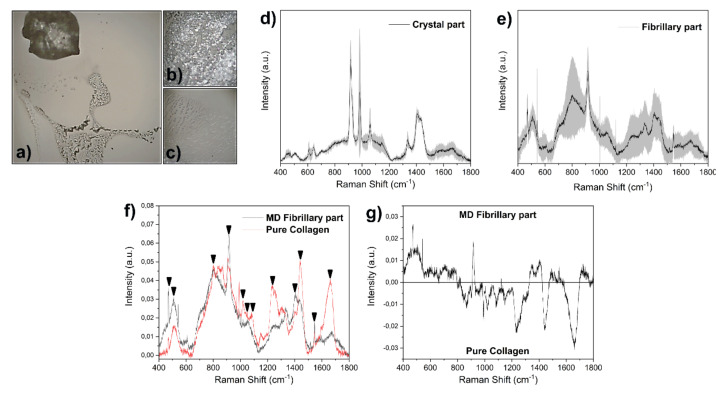
(**a**) Light microscopy micrograph of MD on calcium fluoride (original magnification 50×). In the insets, the crystal (**b**) and fibrillary parts (**c**) at higher magnification (100×) are shown. Raman signals collected from the (**d**) crystal and (**e**) fibrillary parts. The gray bands represent the associated standard deviation. (**f**) Comparison between pure collagen and MD fibrillary part, with peaks of interest highlighted by the black arrows. (**g**) Subtraction spectrum of MD fibrillary part and pure collagen obtained with the error propagation.

Since the morphological analysis was not able to reveal the presence of the coating, we analyzed coated specimens by Raman spectroscopy. As described in the Materials and Methods section, MD and COL were deposited on a calcium fluoride slide and dried overnight [24]. The microscopic analysis revealed two separated regions in MD, characterized by a crystal formation and a fibrillary dispersion (Figure 4a–c). The Raman analysis was focused on these two regions (Figure 4d,e).

The crystal part presents the typical sharp peaks of crystal structures, with peaks attributable to the characteristic signals of riboflavin (750, 1345, 1410 cm^−1^) and ascorbic acid (605 and 632 cm^−1^) (Figure 4d), both present in the MD product [25,26]. The fibrillary part was mainly composed of collagen due to the presence of characteristic peaks at 536, 858, 919, 1065, 1343, 1454 and 1674 cm^−1^ (Figure 4e) [27].

The comparison between COL and the MD fibrillary part (Figure 4f) reveals common peaks at 500, 580, 829, 1248, 1430 and 1650 cm^−1^ with a partial difference in the global spectral shape. A potential explanation can be found in the presence of MD in dissolved salts in the product solution that can alter and modify the conformation, interaction with the environment and structure of the protein. As a consequence, the detected Raman signal is altered, but still consistent with the presence of collagen. The potential attribution of the main peaks (Figure 4f) is reported in Table 1. The main differences between COL and the MD fibrillary part are highlighted by the subtraction spectrum in Figure 4g. The alteration of peaks at 1235 and 1665 cm^−1^ due to the Amide I and III bands and at 1443 cm^−1^ due to the CH3 skeletal deformation indicates a change in the collagen fundamental structure of MD.

### 3.2. Cell Morphology

**Figure 5:**
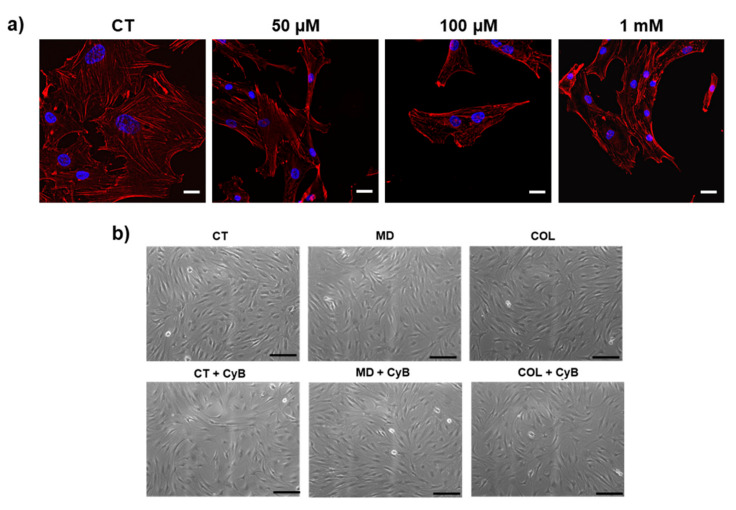
(**a**) Micrographs showing actin filaments detected by rodhamine-phalloidin by THUNDER in control cells (CT) and after administration of CyB at the indicated doses. After 50 μM CyB, actin filaments become shorter and more evident, indicating that the cytoskeleton is not preserved after CyB. (**b**) Representative phase contrast microscopy micrographs showing cell morphology of cells grown on MD and COL, compared to CT. After CyB, CT cells become less flattened and more rounded (Figure 4b). Scale bar 200 µm (**a**) and 20 μm (**b**).

Before analyzing the effect of MD and COL on tenocytes, we first observed the actin cytoskeleton in cells treated with different doses of CyB. Tenocytes possess long microfilaments mostly arranged in longitudinal arrays parallel to the long axis of the cells. At the concentration of 10 µM, CyB is able to block the dynamic instability of the actin cytoskeleton in order to deprive tenocytes of the mechanical stimulation mediated by actin microfilaments. At this concentration, filaments preserved their integrity and their distribution, without any evident morphological modification and without significantly damaging the mechanosensory apparatus. Higher doses strongly induce actin filaments loss, becoming progressively more evident when increasing the dose (Figure 5a). Phase contrast microscopy analysis revealed that cell morphology was unaffected in cells grown on MD and COL, compared to CT. However, when cells are treated with CyB, tenocytes cultured on MD and COL do not change their morphology, while CT cells become less flattened and more rounded (Figure 5b), suggesting that they are less attached to the substrate.

### 3.3. Expression of Genes and Proteins Related to Collagen Turnover

**Figure 6:**
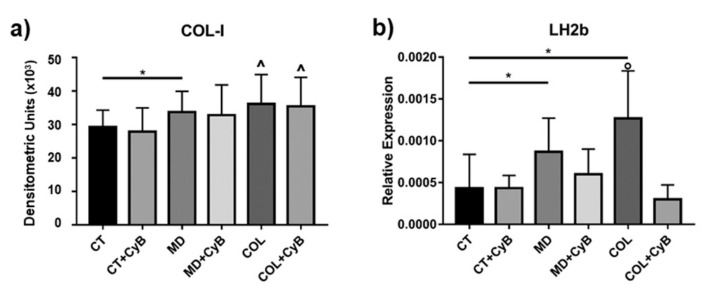
(**a**) Bar graphs showing COL-I protein levels obtained using slot blot after densitometric scanning of immunoreactive bands in the considered experimental conditions. Data are expressed as mean ± SD. (**b**) mRNA levels for Long lysyl hydroxylase 2 (LH2b) in CT and tenocytes cultured on MD and COL with or without CyB treatment assessed by real-time PCR. Data were normalized on GAPDH gene expression and are expressed as mean ± SD for at least two independent experiments. * *p* < 0.05 using *t*-test. ^ *p* < 0.05 vs. CT, CT+CyB, COL+CyB vs. CT+CyB; ° *p* < 0.05 vs. CT and COL+CyB using ANOVA.

COL-I protein levels secreted by tenocytes in cell supernatants were analyzed by slot blot. The statistical analysis using the t-test revealed a significantly increased COL-I secretion in cells cultured on MD (*p* = 0.033) and a trend of increase in cells cultured on COL (*p* = 0.08), compared to CT. CyB administration did not influence COL-I secretion by tenocytes (Figure 6a). The AVOVA *p*-value was statistically significant (*p* = 0.0056) and the post-test showed a significant increase in COL-I in COL vs. CT (*p* = 0.041), in COL vs. CT+CyB (*p* = 0.011) and in COL+CyB vs. CT+CyB (*p* = 0.022).

Collagen maturation was analyzed by assessing the mRNA levels for LH2b, involved in the cross-linking of newly synthetized collagen, by real-time PCR. LH2b mRNA levels were significantly higher in tenocytes cultured on MD and COL (*p* = 0.039 and 0.020, respectively), compared to CT. CyB administration reduced LH2b gene expression in cells cultured on MD and COL (*p* = 0.053 for COL vs. COL+CyB), but not in CT (Figure 6b): this finding suggests that LH2b up-regulation induced by the coating is triggered by a mechanical stimulation mediated by the actin cytoskeleton. The ANOVA p value was 0.0095 and the post-test confirmed the induction of LH2b in COL compared with CT (*p* = 0.025) and revealed a significant decrease in COL+CyB vs. COL (p=0.025).

**Figure 7:**
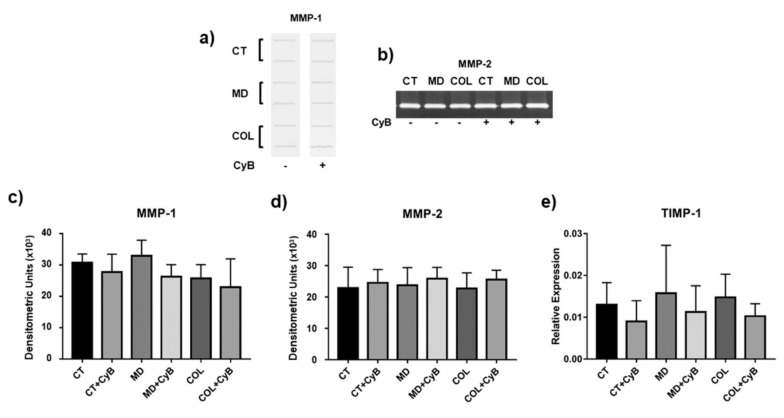
Representative slot blot for matrix metalloproteinase-1 (MMP-1) levels (**a**) and representative SDS-zymography showing MMP-2 activity (**b**) assayed in serum-free cell supernatants of tenocytes cultured in the considered experimental settings. Bar graphs showing MMP-1 protein levels (**c**) and MMP-2 activity (**d**) after densitometric analysis of immunoreactive and lytic bands, respectively. Data are expressed as means ± SD for at least two independent experiments. (**c**,**e**) Bar graphs showing TIMP-1 gene expression after normalization on GAPDH mRNA levels. Data are expressed as mean ± SD for at least two independent experiments.

Interstitial collagen degradation is driven by MMP-1. Slot blot analysis of MMP-1 levels in cell culture supernatants revealed that this collagenase remained unaffected in tenocytes cultured on MD and COL, compared to CT, as well as after CyB administration (Figure 7a,c). A similar pattern of expression was observed for MMP-2 gelatinolytic activity, assessed by SDS-zymography (Figure 7b,d). A similar pattern was also observed for TIMP-1, the main inhibitor of MMP-1, analyzed at the gene expression level by real-time PCR. TIMP-1 mRNA levels revealed wide interindividual differences and were similarly modified by CyB in all the experimental groups (Figure 7e).

### 3.4. Cytoskeleton Arrangement and Vinculin Expression in Focal Adhesions

**Figure 8:**
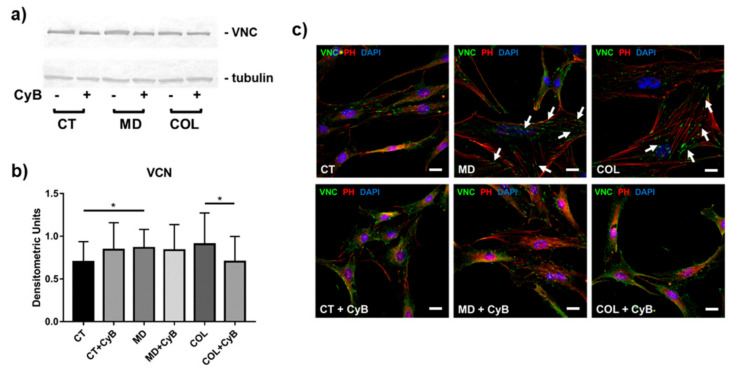
(**a**) Representative Western blot for VNC quantification in cell lysates obtained from tenocytes cultured on MD and COL, compared to CT, with or without CyB treatment. VNC expression was normalized on tubulin. (**b**) Bar graphs showing VCN expression after densitometric analysis of immunoreactive bands. Data are mean ± SD for at least two independent experiments. (**c**). Immunofluorescence analysis for VNC (green) in tenocytes cultured on MD and COL, compared to CT, before and after CyB treatment. Actin filaments are stained using rhodamine-phalloidin labeling. Nuclei are stained in blue by DAPI. Original magnification: 60×. White arrows point to larger and more evident VNC-containing focal adhesions observed in MD and COL samples, compared to the same samples treated with CyB. CyB modified the size of VCN-containing focal adhesions similarly to CT. VNC: vinculin; PH: phalloidin. Scale bar: 20 µm. * *p* < 0.05 using *t*-test.

In order to understand whether MD or COL may represent a mechanical stimulation able to influence the ability of tenocytes to form focal adhesions, we analyzed the expression of VNC, a key protein involved in the formation of the adhesion plaque, by morphological and molecular methods. Western blot analysis showed that VNC protein levels were significantly up-regulated in cells grown on MD (MD vs. CT, *p* = 0.033) and tended to increase also in cells cultured on COL. In this experimental group, VNC was significantly decreased by CyB treatment (COL vs. COL+CyB, *p* = 0.040) (Figure 8a,b). The effects of the presence of the scaffold and of CyB administration were more evident using morphological analysis by immunofluorescence. Indeed, VNC immunoreactivity, localized at the extremities of actin filaments in correspondence with focal adhesion formation on the substrate, was found to be stronger and wider in tenocytes grown on MD and COL, compared to CT (Figure 8c). After CyB administration, the VNC immunofluorescence signal and the regions corresponding to the presence of the focal adhesion seemed less evident and smaller only in cells grown on MD and COL, but not in CT, becoming similar to CT (Figure 8c, arrows).

### 3.5. Wound Healing Assay

**Figure 9:**
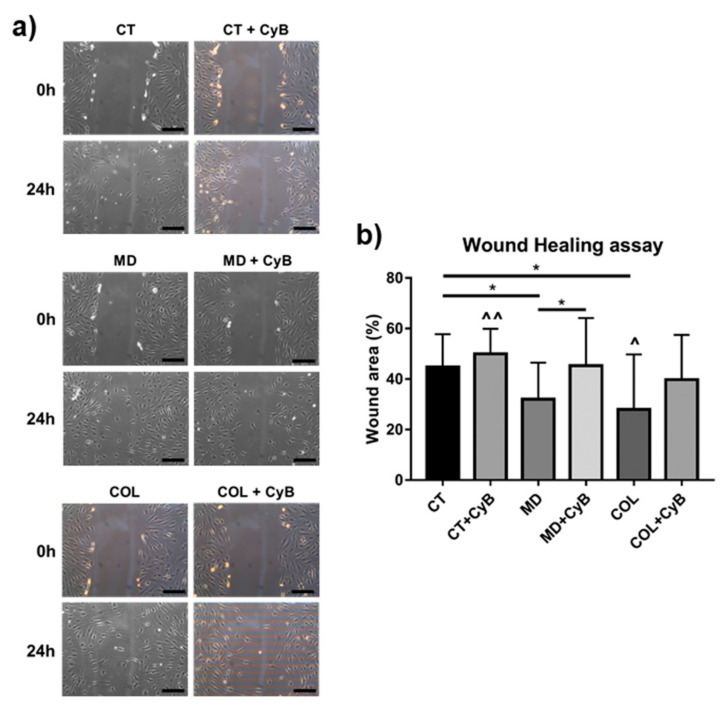
(**a**) Representative phase contrast micrographs showing the results of the wound healing assay in control tenocytes (CT) and tenocytes grown on MD and COL at 0 and 24 h after the scratch, with or without CyB administration. Original magnification: 10×. (**b**) Bar graphs showing the area of wound closure after 24 h, expressed as a % of the area at 0 h, in cultured tenocytes in the different experimental conditions. Data are mean ± SD for at least two independent experiments. * *p* < 0.05 for *t*-test; ^ *p* < 0.05 vs. CT; ^^ *p* < 0.01 vs. MD and COL using ANOVA.

Cell migration, playing a key role during tendon healing, was assessed by a wound healing assay in tenocytes grown on CT, MD and COL with or without CyB administration. The quantification of the scratch size revealed that cell migration is significantly increased in tenocytes cultured on MD and COL, compared to CT (*p* = 0.023 and *p* = 0.032, respectively). Conversely, cell migration remained unaffected by CyB treatment in CT, but was strongly reduced in MD (MD vs. MD+CyB, *p* = 0.040) and, although not statistically significant, in COL tenocytes (COL vs. COL+CyB, *p* = 0.07) (Figure 9a,b). The ANOVA *p* value was 0.001 and the post-test confirmed the increased migration induced by COL compared to CT (*p* = 0.015) and revealed a significant increase in the migration of cells cultured on MD or COL compared to CT+CyB (*p* = 0.009 and *p* = 0.001, respectively).

### 3.6. Expression of FAK, PAX and YAP/TAZ as Mechanosensors

To understand if the scaffold containing MD and COL affects tenocytes biology by a mechanical stimulation, the expression of key proteins playing a role as mechanosensors was analyzed.

**Figure 10:**
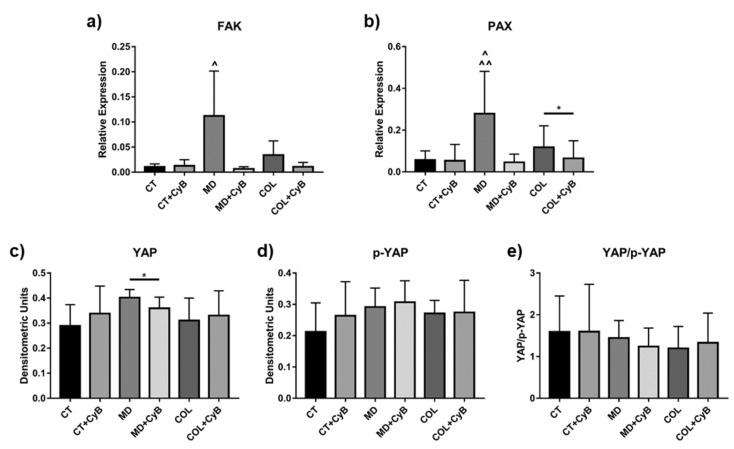
Bar graphs showing FAK (**a**) and PAX (**b**) mRNA levels after normalization on GAPDH gene expression. Data are means ± SD for at least two independent experiments. For YAP expression, the active-form YAP (**c**), the inactive phosphorylated form (**d**) and the YAP/p-YAP ratio (**e**) were assessed by Western blot and represented by the histograms showing mean ± SD for at least two independent experiments for the considered experimental groups. * *p* < 0.05 for *t*-test; ^ *p* < 0.05 vs. CT, CT+CyB, MD+CyB, COL+CyB; ^^ *p* < 0.01 vs. MD+CyB and COL+CyB using ANOVA.

FAK and PAX are proteins in the adhesion plaque that also act as mechanosensors. Their mRNA levels tended to be up-regulated in tenocytes cultured on MD and COL compared to CT, although not reaching the statistical significance (*p* = 0.09). CyB treatment did not affect FAK in CT but had an impact on its expression in cells cultured on MD (*p* = 0.09) and COL, determining its reduction (Figure 10a). The ANOVA revealed that FAK mRNA levels are up-regulated in MD vs. CT (*p* = 0.017) and vs. CT+CyB (*p* = 0.020) and that they are decreased in MD vs. MD+CyB (*p* = 0.013) and COL+CyB (*p* = 0.018). A similar pattern was observed for PAX: its expression was higher in MD (*p* = 0.075) and COL, compared with CT, and was reduced by CyB only in cells cultured on the scaffold (*p* = 0.07 for MD vs. MD+CyB and *p* < 0.05 for COL vs. COL+CyB), whilst it remained unchanged in CT (Figure 10b). The analysis of PAX gene expression by ANOVA showed that its expression was significantly increased in MD vs. CT and vs. CT+CyB (*p* = 0.0059 and *p* = 0.052, respectively), while it was reduced in MD+CyB (*p* = 0.003) and COL+CyB (*p* = 0.007) compared to MD.

Yes-associated protein (YAP) and transcriptional co-activator with PDZ-binding motif (TAZ) are mechanosensors whose activity is regulated by phosphorylation, leading to protein inactivation and cytoplasmic translocation. YAP/TAZ were first analyzed by Western blot using antibodies to detect both the unphosphorylated (active) and phosphorylated (inactive) proteins. YAP and p-YAP resulted in being similarly expressed in cell lysates obtained from CT, MD and COL tenocytes, although a significant down-regulation was observed after CyB administration in cells cultured on MD (*p* = 0.044) (Figure 10c). p-YAP resulted in being similar in all the experimental conditions (Figure 10d) as well as the YAP/p-YAP ratio (Figure 10e). A similar pattern was observed for TAZ and p-TAZ (data not shown).

**Figure 11:**
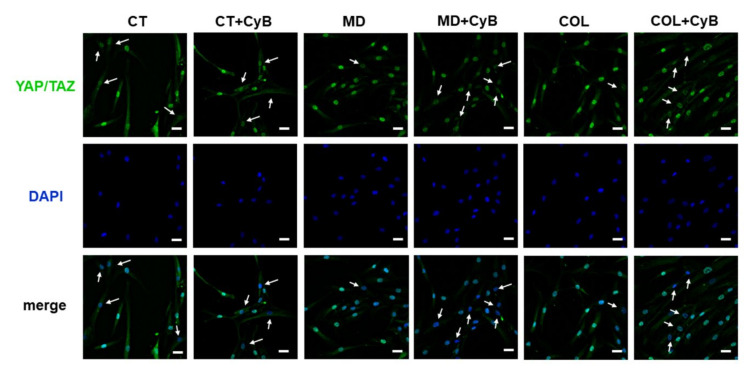
Immunofluorescence analysis for YAP/TAZ (green) in tenocytes cultured on MD and COL, compared to CT, before and after CyB treatment. The merged micrographs show that in tenocytes cultured on MD and COL, immunoreactivity is more evident in the nucleus, while, after CyB treatment and in CT, immunoreactivity is mostly in the cytoplasm and nuclei are more blue, suggesting that the presence of MD and COL induces YAP/TAZ activation, while mechanical stress deprivation induces a phenotype more similar to CT cells. Original magnification: 60×. Scale bar: 20 μm.

In order to understand whether MD or COL were able to trigger a mechanical stimulation in tenocytes, YAP/TAZ activation induced by the scaffold was investigated by analyzing their localization by immunofluorescence analysis. YAP/TAZ were expressed both in nuclei and the cytoplasm. We observed a stronger nuclear immunoreactivity in tenocytes cultured on MD and COL, compared to CT. In CT, CyB did not significantly modify this pattern of expression, whilst in tenocytes cultured on MD and COL, CyB strongly increased the number of nuclei having a less intense YAP/TAZ labeling (Figure 11): this finding suggests that mechanical stimulus deprivation following CyB administration inactivated YAP/TAZ and induced their translocation from the nucleus to the cytoplasm.

## 4. Discussion

The mechanobiology of tenocytes is vital to preserve tendon homeostasis [28,29,30,31]. Tenocytes are able to sense mechanical stimuli imposed on tendons during mechanical loading and can adapt their metabolism in an anabolic or catabolic manner in order to remodel the ECM according to the applied loads [32,33,34]. Therefore, tenocytes are responsible for tendon mechanical adaptation: they convert mechanical stimuli into biochemical signals that ultimately influence tendon adaptive physiological or pathological changes, thus affecting its biomechanical properties [13,15,16]. In fact, it was reported that physiological mechanical loading increases collagen synthesis [14,35], while reduced loading leads to MMP-1 up-regulation [36].

Tensile loading acting on tendons is transduced into intracellular biochemical responses by various sensors and pathways, and the propagation of extracellular-generated forces rely on the actin cytoskeleton [37]. Actin filaments mediate the modification and deformation of the ECM and contribute to the propagation of mechanical stimulation to the nucleus, where gene expression for ECM components can be accordingly affected [38]. It has been demonstrated that the deprivation of mechanical stimulation on tendons mediated by the actin cytoskeleton can be obtained by CyB treatment [36]. Therefore, in order to understand if MD acts as a mechanical scaffold, we utilized CyB to analyze if the effects elicited by MD or COL on tenocytes behavior are affected by mechanical loading deprivation.

For this purpose, we first analyzed the scaffold containing MD and COL at SEM to evaluate if the substrate arrangement could influence cell alignment. The observation at SEM of Petri dishes coated with MD or COL did not reveal the presence of collagen fibrils, possibly due to a fragmentation into small fragments of the collagen contained in the device. As a consequence, when cultured on the scaffold, cells were not influenced in their arrangement and grew without any specific distribution. To support our findings, a further SEM analysis conducted on a commercial Petri dish coated with type I collagen confirmed that collagen fibrils are undetectable. Since, in our previous study, we showed that MD was able to modify some biological activities of tenocytes [19], we tried to demonstrate the presence of the scaffold using a different approach such as Raman spectroscopy. Using this technique, we were able to assess the presence of mainly type I collagen in MD prepared to culture tenocytes.

After demonstrating the presence of the scaffold, we investigated collagen turnover, since COL-I is the main component of the tendon ECM. Its content is regulated by a finely balanced turnover controlled by tenocytes acting at the level of collagen synthesis, maturation and degradation. Collagen turnover, therefore, plays a key role in determining the tendon ability to resist mechanical forces and repair in response to injury [9]. We previously demonstrated that MD favors COL-I secretion [19], suggesting that this medical compound is able to trigger the anabolic phenotype of tenocytes. In the present study, our results confirm the increase in COL-I protein levels in the supernatant of tenocytes cultured on MD and COL, compared to CT. Since CyB administration had no effect on collagen expression in all experimental groups, there is not a clear demonstration that the effect of the scaffold on COL-I expression is mechanically induced and mediated by the actin cytoskeleton.

Maturation of newly synthesized collagen is needed to provide collagen fibril stabilization and tendon tensile strength and is obtained by the cross-linking of newly secreted collagen by enzymes such as LH2b [39,40]. Our results show that LH2b is up-regulated by MD, and also by COL, in tenocytes cultured for 48 h, as previously demonstrated [19]. Interestingly, this effect was lost after CyB administration only in tenocytes cultured on MD and COL, but not in CT, pointing to a mechanical mechanism exerted by MD to trigger collagen maturation to improve collagen stability.

Collagen turnover pathways include collagen breakdown played by MMP-1, which cleaves the intact collagen triple helix, followed by other proteases such as MMP-2 [41,42]. The key role of MMP-1 in tendon ECM homeostasis is based on the previously demonstrated inverse correlation between MMP-1 expression at the gene and protein levels and the amplitude of tensile mechanical load acting on tendons. In fact, low levels of MMP-1 induced by mechanical loading are related to a more stable tendon structure [36]. Here, we show that MMP-1 and MMP-2 levels are not affected by MD and COL, and they remain unchanged by CyB administration. When investigating collagen degradation, TIMPs expression should be also analyzed. TIMP-1 is the main inhibitor of MMP-1, binding MMP-1 in a 1:1 stoichiometric ratio and inhibiting its activation and activity [43,44]. TIMP-1 mRNA levels slightly increased in tenocytes cultured for 48 h on MD and COL, compared to CT, as previously reported [19], and were reduced after CyB administration in all the considered experimental groups. This finding suggests that, in our experimental conditions, TIMP-1 levels are not under specific mechanical control mediated by the actin cytoskeleton. Overall, collagen turnover mechanisms involving the activity of MMP-1, MMP-2 and TIMP-1 seem to be unaffected by CyB.

ECM remodeling and homeostasis are influenced by mechanical stimuli acting on tendons and tenocytes are mechanoresponsive cells: they play a key role as the effectors since they are able to sense mechanical signals and convert them into biological responses [45,46]. This activity of tenocytes is based on their actin microfilaments that represent a mechanotransduction system allowing to adapt tenocyte metabolism in response to different mechanical forces acting on tendons [36]. CyB is known to modify the dynamic instability of actin filaments. However, as shown in Figure 8, the dose of CyB used in this study did not injure microfilaments and tenocytes preserved their structural integrity.

The actin cytoskeleton also plays a key role during cell migration. Since tenocytes migration is needed during tendon healing [47], we investigated, by a wound healing assay, if MD and COL affect cell migration and if their effect relies on a mechanoresponsive mechanism influenced by CyB treatment. We found that MD favors cell migration, as previously reported [19], as well as COL, confirming that the therapeutic activity of this medical device could be related to this effect. To demonstrate that MD-induced cell migration is triggered by a mechanotransduction system, the wound area was measured after CyB administration. Interestingly, CyB was able to decrease cell migration in tenocytes cultured on MD and COL, but not in CT, strongly suggesting that the stimulation of cell migration induced by MD is mediated by a mechanical effect.

During the dynamic process of cell migration, cells undergo a repeated cycle of attachment to the ECM and subsequent detachment of the cell from the matrix. Transmembrane proteins, the integrins, mediate the attachment of tenocytes to the ECM and bridge the inside and outside of the cells. To do this, they link their cytoplasmic domain to the focal adhesion complexes at the leading edge of the cell, including many different proteins such as VNC, a cytoplasmic actin-binding protein enriched in focal adhesions [48,49,50]. Interestingly, the presence of VNC at adhesion complexes is force-dependent [50]. Western blot analysis of VNC revealed some significant modifications induced by the medical device before and after CyB treatment. However, more interesting findings were obtained by morphological analysis using immunofluorescence, which revealed some qualitative differences in cells cultured on MD and COL, compared to CT. In fact, VNC immunoreactivity detectable at the extremity of microfilaments and the size of focal adhesions containing VNC seem more evident and larger in cells grown on the medical device, compared to CT. This observation suggests the hypothesis that VNC expression can be affected by MD and COL, and that the medical devices could improve the attachment of tenocytes to ECM components and, therefore, their ability to form more efficient focal adhesions to favor cell migration. This hypothesis is supported by the observation that, after CyB administration, focal adhesions of cells cultured on MD and COL are similar to those observed in CT. Accordingly, it was reported that VNC recruitment is enhanced when tension increases, while, when tension decreases, focal adhesions are disassembled in response to decreased tension [50]. Moreover, the analysis with the phase contrast microscope revealed that cell morphology was similar in tenocytes grown on CT, MD and COL. By contrast, CyB induced a less flattened morphology in CT, confirming the hypothesis that the medical device is able to favor cell adhesion and thus cell migration.

To finally demonstrate that MD affects tenocyte behavior representing a mechanical stimulus acting on mechanotransduction mechanisms, we analyzed the effect of the medical device on the expression of key mechanosensors such as FAK, PAX and YAP/TAZ. FAK and PAX are components of the adhesion plaque complex. They are involved in the formation of focal adhesions needed for cell migration but they also play a key role acting as mechanosensors [51,52,53]. Our data show that FAK and PAX gene expression is strongly influenced by MD as well as by COL, compared to CT. When CyB is added to the cell culture medium for 48 h, FAK and PAX mRNA levels are down-regulated only in tenocytes grown on MD and COL, and not in CT. This finding suggests that their induction is dependent on the mechanical stimulus exerted by the medical device used as a scaffold. Moreover, this effect is lost when the transmission of the mechanical stimulus on tenocytes is blocked when cells are deprived of their mechanotransduction apparatus. To strengthen this hypothesis, we analyzed the expression of the transcriptional regulators YAP/TAZ, which are regulated by mechanical inputs in a variety of cellular settings, thus impacting many different cell activities [51]. YAP and TAZ act as mechanosensors primarily regulated by the substrate on which cells adhere, which, in turn, influences YAP/TAZ activity stimulating the actin cytoskeleton. The integrity of microfilaments is pivotal on YAP/TAZ activity. In fact, treatment of cells with Latrunculin A, an inhibitor of actin polymerization, results in phosphorylation of YAP and cytosolic localization of YAP/TAZ [51]. In this study, we used CyB to inhibit actin polymerization and to block its dynamic instability in order to analyze YAP/TAZ expression in tenocytes cultured on MD and COL, compared to CT, to demonstrate that the medical device represents a mechanical stimulus to affect cell behavior.

Western blot analysis of YAP/TAZ did not reveal important differences as well as in the YAP/p-YAP ratio. However, our data suggest that MD and COL represent a mechanical input for tenocytes since immunofluorescence analysis demonstrated that YAP/TAZ expression is more nuclear in cells cultured on MD and COL, compared to CT. This suggestion is further supported by the observation that after CyB administration, depriving cells of the mechanical input mediated by the cytoskeleton, YAP/TAZ immunoreactivity becomes less nuclear and more cytoplasmic only in cells grown on MD and COL, and not in CT. This suggestion is consistent with previous studies demonstrating that, since YAP/TAZ serve as mechanotransducers and mechanosensors, their subcellular localization and activity are tightly regulated by cell substrate rigidity and tensile inputs from the ECM [53,54,55], and that cytoskeletal tension is required for YAP/TAZ nuclear localization [53].

## 5. Conclusions

Considered as a whole, these in vitro findings suggest that MD and COL trigger similar responses in tenocytes and that their effect on tenocytes behavior represents a mechanical input involving the mechanotransduction machinery. In particular, we showed that MD-Tissue influences some tenocytes activity involved in ECM homeostasis and improves focal adhesion formation and migration ability. Overall, we confirm that MD-Tissue, acting as a mechanical scaffold, could be an effective medical device used as a novel therapeutic, regenerative and rehabilitative approach to favor tendon healing in tendinopathies.

## Figures and Tables

**Table 1:** Potential peaks attribution, based according to Carcamo et al. [23].

Raman Shift (cm^−1^)	Attribution
475	Skeletal deformations
508	Skeletal deformations
800	Skeletal C-C vibrations
920	C-COO^−^ vibrations
992	Phenylalanine
1015	Vibration of Proline C-N
1055	Distortion of Proline N-C-H
1235	Amide III
1403	Deformation of CH_3_
1443	Deformation of CH_3_
1544	Deformation of NH_3_^+^
1665	Amide I
